# Coping Processes of Congolese Refugee Women Newly Resettled in the United States: A Qualitative Exploration

**DOI:** 10.3390/ijerph22081208

**Published:** 2025-07-31

**Authors:** Na’Tasha Evans, Kamesha Spates, Cedric Mubikayi Kabasele, Chelsey Kirkland

**Affiliations:** 1 Department of Behavioral Science, College of Medicine, University of Kentucky, Lexington, KY 40536, USA; 2 Department of Africana Studies, University of Pittsburgh, Pittsburgh, PA 15260, USA; kspates11@pitt.edu; 3 Department of Epidemiology, College of Public Health, Kent State University, 800 Hilltop Dr., Kent, OH 44242, USA; cmubikay@kent.edu; 4 Center for Public Health Systems, University of Minnesota School of Public Health, 420 Delaware St SE, Minneapolis, MN 55455, USA; ckirk@umn.edu

**Keywords:** refugees, Congolese, women, coping, resettlement

## Abstract

The present study aimed to provide Congolese refugee women with an opportunity to narrate firsthand experiences coping with resettlement challenges in the United States. Translator-assisted, face-to-face semi-structured individual interviews were conducted with newly resettled Congolese refugee women (n = 20) aged 18 and older who arrived in the United States between 2011 and 2018. All participants were receiving assistance from a resettlement agency, located in the Midwestern US, at the time of the study. Data were analyzed using descriptive coding and thematic analysis. Three overarching themes were developed, indicating that Congolese refugee women adopt three main coping mechanisms to deal with challenges they face after resettling in the United States: (1) use of social support, (2) acceptance of the situation, and (3) spirituality. Resettlement support services, such as those provided by resettlement agencies, mental health providers, and community-based organizations, should integrate both economic and cultural dimensions into their services to address the complex physiological, mental, and emotional impacts of resettlement. These services should prioritize culturally and spiritually sensitive techniques that are linguistically accessible.

## 1. Introduction

The United Nations High Commissioner for Refugees (UNHCR) defines refugees as individuals who have been forced to flee their country and seek safety beyond its borders because of persecution, war, violence, and/or human rights violations [1]. Although the United States has steadily reduced the number of accepted refugees in recent years, it remains the largest refugee resettlement destination in the world, with approximately three million refugees resettled since the Refugee Act of 1980.

Over the past decade, many refugees have been resettled from the Democratic Republic of the Congo [2]. The Democratic Republic of Congo, the second-largest country in Africa, has been mired in armed conflicts for decades [3,4]. The conflict in its eastern region, dubbed the deadliest since World War II, has resulted in over six million casualties since 1996, as well as millions of refugees being internally displaced [5,6].

Congolese women in conflict zones have endured a widespread occurrence of sexual and gender-based violence [7,8,9]. Throughout the Democratic Republic of Congo, such violence has been systematically used as a weapon of warfare to penalize civilian populations for their perceived support of opposing factions [10,11].

Previous studies have examined the journey and arrival of Congolese refugees in the United States through surveys and other innovative methods, such as photovoice. Additionally, some have investigated the influence of Congolese refugee women’s familial beliefs and cultural identity on their resettlement experiences in the United States. We aim to build on this research by exploring the coping strategies these women employ through first-hand accounts. Therefore, this study aims to gain a better understanding of Congolese refugee women’s firsthand experiences with coping during resettlement upon arrival in the United States. 

## 2. Literature Review

### 2.1. Congolese Refugee Women’s Experiences Before Resettlement in the United States 

Prior to resettling in the United States, many Congolese refugee women lived in refugee camps with their children to flee persecution [3,4]. In the refugee camps, they often faced challenges such as violence, structural discrimination, food and water shortages, poor health, and sanitation problems [12,13,14]. Furthermore, because of the separation from family members and the disruption of social structures caused by armed conflicts, up to forty percent of Congolese women have experienced sexual violence and trauma as refugees [15,16] through forced prostitution, sexual exploitation in return for access to resources and essential needs, domestic violence, and sexual attacks or coercion, including by camp authorities and workers [9,17].

All the problems that Congolese refugee women face while living in camps are exacerbated by the length of their stay [18]. Refugee camps, in nature, are designed to provide temporary housing while waiting to return to their homes if deemed safe or being resettled to a permanent location. However, most Congolese refugees live in a protracted refugee situation, defined by the UNHCR as “one in which refugees find themselves in a long-lasting and intractable state of limbo. Their lives may not be at risk, but their basic rights and economic, social, and psychological needs remain unfulfilled after years in exile” [18]. Usually, this happens when refugees are displaced for more than five years [19]. Congolese refugee women in our study have reported living in refugee camps for up to 20 years before resettlement.

### 2.2. Mental Health Risks and Sequelae of Trauma

Twenty percent of Congolese refugee women in the United States are referred to as “women-at-risk”, which is defined as women or girls who experience reduced protection based on their gender and lack effective protection normally provided by male family members, face compounded challenges due to the intersection of gender, limited kin networks, and cultural displacement. These women may be single, accompanied, or unaccompanied by other family members. Upon migrating to the United States, the challenges faced by these at-risk women become exacerbated due to their novelty in the environment, restricted familial connections, and insufficient understanding of how to navigate their new lives within the United States [14,20,21,22]. Previous research noted that integration difficulties may influence how they cope with resettling in the United States [23].

### 2.3. Economic Integration 

Upon arrival in the United States, refugees receive support, such as food, clothes, housing, language training, and employment guidance from refugee agencies [24]. The United States Refugee Resettlement Program has emphasized assisting newly resettled refugees to obtain a job as soon as possible for them to achieve economic self-sufficiency by [24,25], expecting refugees to obtain a job and/or enroll for social benefits after the initial assistance from refugee agencies expires [26]. However, finding employment is a challenge for Congolese refugee women as most do not speak English, lack previous work experience, and/or formal education [27,28]. Thus, they often end up in low-paying jobs with little opportunity for occupational advancement [23,27,28,29,30]. Furthermore, Congolese refugee women who have children but no one to help take care of them are faced with difficulty keeping their employment. Consequently, many are forced to choose between caregiving and economic survival, reinforcing economic dependency and social isolation [31,32].

### 2.4. Mental Health and Coping with Trauma, Including Barriers to Health Care 

Refugees are required to receive a comprehensive health screening overseas to be cleared for admission into the United States and then receive a subsequent domestic medical screening upon arrival [33,34]. Most refugees including Congolese women are coming to new communities after spending years in refugee camps or poverty-stricken and conflict-torn regions [20,35,36], and many are torture or sexual assault survivors and/or are affected by undiagnosed or untreated chronic conditions [33,37]. Due to cultural and ethnic differences between refugees and health care providers, it can be difficult for refugees to clearly explain their experiences and understand a provider’s diagnosis, even with the help of a translator due to stigma, religious beliefs, and the fear of traumatization [38,39,40].

### 2.5. Cultural and Linguistic Integration

The UNHCR defines the integration of refugees as “a dynamic and multifaceted two-way process which requires efforts by all parties concerned, including a preparedness on the part of refugees to adapt to the host society without having to forego their own cultural identity, and a corresponding readiness on the part of host communities and public institutions to welcome refugees and meet the needs of a diverse population [1]”. Furthermore, the process of integration is complex and gradual, comprising distinct but interrelated legal, economic, social, and cultural dimensions, all of which are important for refugees’ ability to integrate successfully as fully included members of the host society [22]. Cultural integration is the process through which immigrants adopt the essence of another culture while at the same time maintaining their own culture [41].

Refugees often experience stress due to the expectations of cultural integration, language adjustment, and substantial differences in social structures, norms, and values compared to their country of origin [42]. Language barriers are one of the largest barriers that refugees face after resettling in the US, especially since resources for social, economic, and cultural integration are closely related to the level of English literacy [38,43]. Congolese refugee women are particularly affected because they have a lower literacy level in their native language due to the limited access to education in refugee camps for women and girls, thus increasing their vulnerability in resettlement. Oftentimes, their education is hindered by cultural factors such as early/forced marriage and parents’ lack of interest in female education [28,44]).

#### Current Study

A gendered and intersectional understanding of Congolese refugee women’s experiences is essential to effective and efficient support systems. Understanding how gender norms, family, socioeconomic status, and religion intersect with the vulnerabilities of resettlement is important. While refugee integration has received increased attention in recent years, the lived experiences and coping strategies of Congolese refugee women remain underexplored in both research and practice. Given their unique cultural, gendered, and economic challenges, a deeper understanding is essential to understanding the complexities of this population’s experiences. The current study provides Congolese refugee women with an opportunity to narrate firsthand experiences with coping during resettlement in the United States. Our research question was as follows: How do Congolese refugee women cope with stressors after resettling in the United States?

## 3. Methods

### 3.1. Study Design

This descriptive qualitative study used semi-structured individual interviews. We used this methodology as it provided the interviewers with the flexibility to gather in-depth, contextual stories of the participants’ firsthand experiences with coping during resettlement [45,46,47,48]. Additionally, the researchers determined richer data would be gathered through dialog between the participant and researcher rather than through interactions between participants [48].

### 3.2. Participants

Twenty women were interviewed for our study. The eligibility criteria were (1) self-identification as a Congolese refugee, (2) self-identification as a woman, (3) be 18 years of age or older, (4) reside in Ohio, and (5) arrived in the United States in 2010 or later. These eligibility criteria were set to provide Congolese refugee women an opportunity to narrate firsthand experiences with coping during resettlement in the United States. Therefore, we wanted participants to have been resettled long enough to have had numerous experiences, while still being recent enough to remember with high accuracy their various coping situations and processes.

### 3.3. Data Collection

The Institutional Review Board at Kent State University (protocol code 18-198 and 22 May 2018) approved this study. Recruitment flyers were posted and distributed at a partner resettlement agency. Those interested in participating contacted a translator. The translator screened all interested parties for eligibility and then signed up all eligible participants for open interview time slots. Interviews were conducted in a private room within the resettlement agency. A translator, an interviewer, and at least one research assistant were present at each interview. Immediately before each interview, verbal informed consent was audio recorded and obtained by the translator. Additionally, the research team had a member from the Democratic Republic of Congo on the team who could verify the verbal informed consent. Lastly, once informed consent was taken, the translator signed a document, agreeing that the verbal consent was provided, and the audio recording was acceptable. Authors NE or KS then provided an explanation and purpose of the study, asked participants to complete a demographic questionnaire, answered participants’ questions about the study and procedure, and then reminded each participant that their answers would not be shared with the resettlement agency, the interview would not affect their current or future care, and that their interview would be audio recorded for transcription. The interview was then initiated and audio-recorded by one of the co-conducting authors, NE or KS. NE or KS would pose the interview questions, which were then translated into the participant’s native language. The participants’ responses were subsequently translated back to English. Each interview lasted about one hour, after which each participant received a USD 10 cash incentive for their time.

### 3.4. Measures

The demographic questionnaire was completed before the start of each interview asked the following: participant age, employment status, whether they spent time in a refugee camp (s), number of refugee camps, length of time in a refugee camp (s), location of refugee camp (s), arrival to the United States, and who came with them to the United States. A semi-structured interview guide was developed based on reviews of the literature. Participants were asked the following question: (1) How do you cope with these problems from resettling in the United States? Since the guide was semi-structured, this question was followed up with an additional probing question. The probing question was, “Who do you talk with about these problems?”

### 3.5. Research Team Positionality Statement

The first and second authors are Black women born in the United States with doctorate degrees and extensive research backgrounds examining health disparities and minority health. The third author holds an MPH, is a Democratic Republic of Congo native, and has a background in health disparities and epidemiology. The fourth author is a white woman born in the United States who holds a doctorate in public health and has a background in health disparities and social determinants of health. Together, their experiences shape their world views and perceptions. During this research, the researchers checked in with each other to ensure the participants’ experiences were at the forefront and mitigated the incorporation of any personal biases.

### 3.6. Data Analysis

To ensure accuracy, author CM, a native of the Democratic Republic of Congo, reviewed all audio recordings to verify the fidelity of the translation. Authors CK and CM transcribed verbatim all audio files and coded each using descriptive coding and thematic analysis [45,46,47,48] using QSR International’s NVivo 12 Pro qualitative data analysis software. Author NE reviewed each theme for accurate representation within the data, and we used intercoder reliability to finalize each theme. The first round of coding used descriptive coding with thematic analysis for the second and subsequent rounds. Descriptive coding assigns labels to data using a word or short phrase to summarize, which was appropriate for this social study [45,46,47,48]. Lastly, we used thematic analysis, which is appropriate for use within the exploration of participants’ psychological world of beliefs, constructs, and emotional experiences. While the translator aimed to convey participants’ responses verbatim and as accurately as possible, certain emotional expressions, words, or cultural concepts may not have direct equivalents in English. This can result in the loss of affective tone or cultural context. To mitigate this, the research team engaged in reflective discussions during data analysis. Additionally, author CM’s review of the original audio recordings allowed for detection of phasing that may not have been fully captured in translation.

## 4. Results

### 4.1. Characteristics of Participants

A total of 20 women aged between 19 and 68 were interviewed (Table 1). The participants had resettled to the United States between 2011 and 2018, with most (n = 17) arriving in 2016 or later. Most (n = 16) spent some time in one or more refugee camps before traveling to the United States, and they all resettled with at least one other family member. Only two participants were employed at the time of the interview.

### 4.2. Qualitative Results

We identified three overarching themes when analyzing the coping processes of Congolese refugee women newly resettled in the United States: (1) use of social support, (2) acceptance of the situation, and (3) spirituality. Please refer to Table 2 for a list of themes and corresponding subthemes, definitions, frequency, and supporting quotes.

#### 4.2.1. Theme 1: Use of Social Support 

A total of twelve participants identified the use of social support they received, both informally and formally, particularly from family and resettlement agencies, as a major factor in coping with the challenges of resettling in the United States. Interview data coded in this category indicated words or phrases that highlight specific language that underscore the importance of turning to others and understanding how social networks impact support with resettling in the United States.

Family members, including children and spouses, play a critical role in the participant’s coping process, often through financial contribution or constraints. Several participants emphasized a shared sense of economic responsibility. However, sometimes, support was not always freely given. For example, a 44-year-old participant stated, “…and also hoping that my children will get jobs too. So that uh we’ll be able to put together all the money we are getting, support one another, pay all our bills. And that will make it much easier for me and my family”. This quote illustrates how mutual financial support within the family was not only a coping strategy but a source of hope and stability. However, sometimes, support was not always freely given. A 68-year-old participant described how an external intervention led to increased familial support, “…before—the case worker talked to my son about his behavior and not contributing in the house, before he was not paying a penny. But after that, he started contributing, I would pay half and he would pay half, but he was not happy about that”. This statement elucidates the tension that can exist within familial support systems. Another participant (48-year-old) recounted the strain on household resources, saying “My husband alone couldn’t support the family and my child had to drop out of school to help out”. This account underscores how economic burden can disrupt family roles and educational aspirations.

In addition to familial support, participants frequently mentioned the vital role of the refugee resettlement agency. The refugee resettlement agency provided immediate relief and longer-term assistance, helping to stabilize participants’ early experiences. For example, a 35-year-old participant stated, “Here they helped me. I left my home, but here they helped me. There are people here who helped me, giving me cash assistance and connecting me to social security”. Likewise, a 48-year-old participant stated, “The only thing we know is we get help from this organization”. Another participant (42-year-old) added, “The problem I had at my house was, um, for us not having a job, we were not able to pay for rent, and we came here to the office, and they helped us”. While the theme level of support emerges as central to participants’ coping processes, the accounts also suggest a complex interplay between support systems and the dual function of resettlement agencies as both safety nets and bridges to important services.

#### 4.2.2. Theme 2: Acceptance of the Situation

A total of ten participants described acceptance of their situation as a significant coping strategy. Interview data coded in this category included words or phrases that highlighted contrasting perceptions among the participants, hopelessness, and perseverance, revealing a dynamic tension between resignation and resilience in response to challenges of resettlement. When assessing acceptance of the situation, participants reported feeling discouraged and at times hopeless, particularly about their financial standing. For example, a 48-year-old participant stated, “Now what can we do? We can’t just think about these things; we have to live like Americans. Sometimes, we don’t have enough to pay the bills, it may be a month, and we can’t afford the bills, so it is difficult. But we are trying our best to pay the bills even when we pay late”. This quote reflects a form of pragmatic acceptance, in which the participant acknowledges ongoing hardship while attempting to conform to perceived cultural norms of resilience. Another participant (55-year-old) added, “…so that is what I struggle with, and I try to bear it because there is nothing I can do about it”. In this quote, acceptance emerges not from optimism but from a perceived lack of alternatives. Likewise, a 30-year-old participant said, “…and sometimes it is too much so I cry. I ask myself if I should go back, yet I know I can’t because I ran away from death. It is just like that; it is very difficult”. This statement illustrates how accepting one’s circumstances may be accompanied by grief, fear, and emotional exhaustion. Although returning is not an option, the pain of adjusting to a new reality is overwhelming.

Further, when examining acceptance of the situation, participants also highlighted the importance of persevering despite their predicament. As a 30-year-old participant stated, “Sometimes, I persevere, and sometimes it is too much, so I cry”. Similarly, a 48-year-old participant added, “The only thing we know is we get help from this organization, the rest is perseverance”. Another participant (64-year-old) stated, “I just do everything by myself, but I wish my children were here because they would be helping me to wash my clothes and cook for me”. These expressions underscore the emotional sacrifices embedded within acceptance. While participants may appear to have accepted their circumstances, their narratives reveal a deeper sense of loss, loneliness, and unfulfilled expectations. Overall, this theme illustrates how acceptance is not a passive state but a complex and often painful coping strategy that enables participants to function in adversity.

#### 4.2.3. Theme 3: Spirituality

A total of four participants reported relying on their spiritual side to cope with resettlement. Interview data coded in this category indicated words or phrases that highlighted the participants’ ability to turn inward and use their faith for inner peace and well-being.

For these participants, spirituality provided an internal anchor. A 55-year-old participant stated, “What I have done is to give everything to God. Because before I had problems, he was there to help me cope with those problems. And I know even now he’s still going to be there for me to help me until the next time”. This statement reveals a deep sense of spiritual continuity. Faith was not only retrospective but also offered reassurance in an uncertain future. Similarly, a 54-year-old participant said, “What helps me is prayer, where I ask God to help me and get me out of the problems. God has helped me. I thank God”. Here, prayer functions as an active coping mechanism. It demonstrates a sense of being seen and supported. In addition, a 58-year-old participant added, “What usually helps me is to pray to God”. While fewer participants explicitly referenced spirituality, those who did emphasized its central role in coping during resettlement.

## 5. Discussion

To provide Congolese refugee women an opportunity to narrate firsthand experiences with coping during resettlement in the United States, results indicated that (1) the use of social support, (2) acceptance of the situation, and (3) spirituality were recurrent themes.

When using social support, participants reported that family members and refugee resettlement agencies helped them cope with resettlement by providing financial, emotional, and logistical assistance. This finding was consistent with the literature. The United States government collaborates with voluntary organizations, including faith-based groups, to support refugees throughout the resettlement process. These organizations ensure that refugees have shelter, food, and basic services in the first 30–90 days in the U.S. and beyond [39]. They also assist with language learning, access to health care, and finding employment [13]. For many refugees, like refugee Congolese women, these organizations replace social networks they were forced to abandon after leaving their countries [32,49]. In addition to the help they received from these organizations, our findings were consistent with the literature as participants also obtained help from family members who were already established in the U.S. [49].

Further, participants reported that acceptance of their situation helped them cope. Participants reported feeling helpless with the situation they were in. This feeling can be explained as an emotional numbing and maladaptive passivity that is induced through repeated (failed) attempts to escape a difficult situation. Participants in this case conclude that their fate is out of their control. 

Additionally, participants reported relying on spirituality to cope with resettlement. These findings are consistent with the literature [13], that refugee Congolese women relied on prayers as a major coping mechanism when faced with challenges both pre- and post-resettlement. These findings are also consistent with literature that has found that refugees, specifically Congolese refugee women, trust in their faith in God to give them strength when faced with adversity [50,51,52,53,54].

### 5.1. Contributions to the Literature 

The work of Evans and colleagues [20] highlights the plethora of challenges faced by Congolese refugee women resettling in the United States in hopes of providing empirically based knowledge to better support these women. This study adds to a very limited body of literature on the experiences of Congolese refugee women in the United States by focusing exclusively on how Congolese refugee women cope with these challenges. This paper contributes to the literature in several ways. First, this study provides Congolese refugee women with an opportunity to narrate firsthand experiences with coping during resettlement in the United States. Secondly, this study focuses solely on Congolese refugee women. We center the Midwestern U.S., thereby capturing a critical yet underexamined geographic and temporal context. Furthermore, we translate participants’ voices into practical recommendations for resettlement agencies and case workers.

### 5.2. Practice Implications

With the number of resettled Congolese refugee women increasing in the United States in recent years, especially in Ohio, the study location is highly relevant. Assessing Congolese refugee women’s firsthand experiences with coping during resettlement in Ohio may assist with gaining a better understanding of their coping mechanisms to provide critical information for professionals (e.g., case workers, health care providers, etc.), resettlement agencies, and community and faith-based organizations for improved assistance, increased funding, supplemental resources, spirituality, resiliency, and the ability to strengthen the family connection. Further, by understanding Congolese refugee women’s firsthand experiences with coping, supportive measures and networks could be implemented to better serve and assist in the resettlement process and with acceptance of the situation.

Economic integrations should be incorporated into services to address the complex physiological, mental, and emotional impacts of resettlement. These services should emphasize linguistically accessible, culturally sensitive techniques that are spiritually informed. Those who are resettling should be empowered through parenting courses, literacy (health and financial) classes, employment programs, and spirituality and support groups.

Lastly, although the women participating in this study did not explicitly disclose their histories of trauma, it is essential for future scholarly inquiries involving Congolese refugees to consider the social and political context antecedent to their migration to the United States. The literature notes that these women encounter challenges upon their arrival that are only compounded [14,20,21,22]. Considering that these women sometimes migrate with their families, it is also essential to consider the potential benefits of providing support not only to the women but also to their familial unit.

### 5.3. Limitations

Although our study had numerous strengths, it also had some limitations. First, our research team concentrated exclusively on Congolese refugee women residing in the state of Ohio. This narrow focus may not adequately represent the broader experiences of Congolese refugee women across various regions of the United States. Therefore, future research endeavors should aim to broaden the geographical scope to encompass women from diverse locations nationwide. The decision to interview participants at the resettlement agency was motivated by safety concerns and the transportation challenges faced by Congolese refugee women. However, it is essential to acknowledge the potential impact of conducting interviews with Congolese refugee women at a resettlement agency. This limitation prevented us from engaging with women who were not directly associated with the agency, potentially introducing a selection bias. We also acknowledge that the women could have been reluctant to share certain things out of fear that the resettlement agency might reprimand them. Furthermore, the interviews did not specifically ask the Congolese refugee women about how they cope with the trauma and sexual violence; thus, the study does not allow us to speak to these experiences. Lastly, the participant narratives were provided to us through a translator, who was also an employee of the resettlement agency. Although we had a research team member fluent in the native language of the participants, we acknowledge that some meaning nuances (e.g., language and cultural references) may have been lost in translation. As such, future research could benefit from collecting the narratives of Congolese refugee women in their native language, thereby enhancing the authenticity and richness of the data collected.

## Figures and Tables

**Table 1:** Demographic characteristics of participants (*N* = 20).

Characteristics	%	*n*
How long have you been in the United States?		
2011	5%	1
2015	10%	2
2016	45%	9
2017	5%	5
2018	3%	3
What is your employment status?		
Not working	90%	18
Full-time	10%	2
Did you spend time in a refugee camp?		
No	20%	4
Yes	80%	16
How many refugee camps were you in?		
0	20%	4
1	50%	10
2	25%	5
3	5%	1
Where were the camps located? Where did you go as a refugee?		
Uganda	25%	5
Rwanda	40%	8
Burundi	10%	2
Tanzania	20%	4
Multiple Locations	5%	1
Who did you come with?		
Self	10%	2
Child	5%	1
Multiple children	20%	4
Husband and child or children	40%	8
Sibling	10%	2
Sibling and child or children	5%	1
Parent(s) and child or children	5%	1
Parent(s), sibling(s), child or children	5%	1

**Table 2:** Themes, definitions, frequency, subthemes, and supporting quotes.

Theme	Definition	Frequency of Theme N (%)	Supporting Quotes
Use of Social support	Words or phrases that highlighted the support Congolese refugee women received from their social networks that impacted their coping processes with resettling in the United States	12 (60%)	“…So now it is only my husband that is working and that is the only income that we have” 55-year-old. “I know my case worker has been really trying her best for me”. 55-year-old “If a problem comes up, I can inform my case worker about the problem”. 48-year-old
Acceptance of the situation	Words or phrases that highlighted the participants’ helplessness and perseverance	10 (50%)	“It’s a problem but what can I do? I just sit. When they tell me to do this and that, I do only what I can and leave what I can’t”. 28-year-old “Sometimes, I persevere, and sometimes it is too much so I cry” 30-year-old
Spirituality	Words or phrases that highlighted the participants’ faith and hope for inner peace and well-being	4 (20%)	“With all these problems I usually depend on God. Because I know I, he’ll keep opening new doors for me”. 55-year-old

## Data Availability

The data supporting the findings of this study are not publicly available due to privacy and ethical considerations. However, the corresponding author will provide access to the data upon reasonable request.
